# The Effect of *Lactobacillus plantarum* ATCC 8014 and *Lactobacillus acidophilus* NCFM Fermentation on Antioxidant Properties of Selected *in Vitro* Sprout Culture of *Orthosiphon aristatus* (Java Tea) as a Model Study

**DOI:** 10.3390/antiox1010004

**Published:** 2012-09-26

**Authors:** Dase Hunaefi, Divine N. Akumo, Heidi Riedel, Iryna Smetanska

**Affiliations:** 1 Department Method in Food Biotechnology, Institute of Food Technology and Food Chemistry, Berlin University of Technology, Königin-Luise Str. 22, 14195 Berlin, Germany; E-Mails: heidiriedel80@yahoo.de (H.R.); smetanska@mailbox.tu-berlin.de (I.S.); 2 Department of Food Science and Technology, Bogor Agricultural University, Bogor, Indonesia; 3 Institute of Biotechnology, Laboratory of Bioprocess Engineering, Berlin University of Technology Ackerstr. 71-76, 13355 Berlin, Germany; E-Mail: akumo2@yahoo.com; 4 Department of Plant Food Processing, University of Applied Science Weihenstephan-Triesdorf, Steingruber Str. 2, 91746 Weidenbach, Germany

**Keywords:** antioxidant, *Orthosiphon aristatus*, fermentation, *in vitro* sprout culture

## Abstract

High rosmarinic acid (**RA**) productivity has been achieved by applying jasmonic acid and yeast extract elicitors to the *in vitro* sprout culture of *Orthosiphon aritatus *(**IOSC**). The highest RA accumulation from three solvents was detected in IOSC after treatment with yeast extract (5 g/L). HPLC analysis clearly confirmed a drastic increase in RA subjected to yeast extract elicitation. Therefore, this yeast extract elicited IOSC was chosen for a lactic acid bacteria (**LAB**) fermentation study as a model system. This selected IOSC was subjected to different types of LAB fermentations (*Lactobacillus plantarum* ATCC 8014 and *Lactobacillus acidophilus *NCFM) for different periods of time 24, 48 and 72 h. The LAB fermentations consisted of solid state fermentations (**SSF**) and liquid state fermentations (**LSF**) in a Digital Control Unit (**DCU**) fermenter system. The aim was to determine the effect of fermentation on the antioxidant properties of the plant extract. Results indicated that all types of LAB fermentation decreased the level of RA and total phenolics, however, a slight increase in total flavonoids and flavonols was observed in SSF samples. HPLC results confirmed that the longer the fermentation, the greater the reduction in RA content. The highest reduction was obtained in the sample of LSF inoculated with *L. plantarum* for a period of 72 h. The temperature of fermentation (37 °C) was predicted as contributing to the declining level in RA content. The loss in RA was concomitant with a loss of total antioxidant activity (1,1-diphenyl-2-picrylhydrazyl (**DPPH**) scavenging activity, Trolox Equivalent Antioxidant Capacity (**TEAC**), and Superoxide Dismutase (**SOD**)-like activity). These results indicate that RA is the major contributor to the antioxidant activity of this plant.

## Abbreviations

RA: Rosmarinic Acid
IOSC: *in vitro *Sprout Culture of *Orthosiphon aristatus*
LAB: Lactic Acid Bacteria
SSF: Solid State Fermentations
LSF: Liquid State Fermentations
LP: *Lactobacillus plantarum*
LA: *Lactobacillus acidophilus*
OD: Optical Density
LP-SSF: Solid State Fermentations inoculated with *Lactobacillus plantarum*
LA-SSF: Solid State Fermentations inoculated with *Lactobacillus acidophilus*
LP-LSF: Liquid State Fermentations inoculated with *Lactobacillus plantarum*
LA-LSF: Liquid State Fermentations inoculated with *Lactobacillus acidophilus*
DPPH: 1,1-diphenyl-2- picrylhydrazyl
TEAC: Trolox Equivalent Antioxidant Capacity
SOD: Superoxide Dismutase
JA: MS medium with Jasmonic Acid
YE: MS medium with Yeast Eextract
TE: Trolox Equivalent
GAE: Gallic Acid Equivalent
QE: Quercetin Equivalent
DW: Dry Weight

## 1. Introduction

Rosmarinic acid (**RA**) is an ester of caffeic acid and 3, 4-dihydroxyphenyllacticacid. It is commonly found in plants from the *Lamiaceae* family, e.g., *Orthosiphon aristatus*, and *Coleus blumei*. This caffeic acid is one of the most important and targeted phenolic compounds in formulating functional food and supplements due to its interesting biological activities, such as antiviral, antibacterial, anti-inflammatory and antioxidant [1]. 

RA from medicinal plants such as *Orthosiphon aritatus* that contain naturally occurring antioxidants is preferable [2]. *Orthosiphon aristatus* is a native tropic-medicinal plant from Southeast Asia. It belongs to the *Lamiaceae* family. RA was identified as the most abundant polyphenol (caffeic acid derivative) in *O. aristatus* and it was thought to be the main contributor for the therapeutic effects and antioxidant activity of this plant [3]. The escalating demand of RA led us to conduct this research in order to increase the production of RA in *Orthosiphon aristatus* and to characterize the antioxidant properties of this plant extract, which may provide useful information for better formulation of the application of functional foods.

Indeed, plant cell cultures that accumulate RA have been proposed for production of this phenolic compound [2]. Plant tissue cultures using plantlets that were obtained from direct shoot organogenesis are generally preferred for mass production of the targeted compound as they are more genetically stable [4]. Moreover, as a native tropic-medicinal plant, to grow *Orthosiphon aristatus* in a sub-tropic climate area has been considered to be relatively difficult. Therefore, *in vitro* sprout culture of *Orthosiphon aristatus* (**IOSC**) was used as the media for this study.

Other reasons for using IOSC are (1) this *in vitro* system may easily control biotic and abiotic stresses on the cultivar, (2) this system allows the harvest of this plant under any conditions regardless of climate condition and seasons, (3) and also through this system, rapid growth mass production can be achieved as well as the combination with sustainable secondary metabolites production [4,5]. Furthermore, this system can also be used to facilitate the fermentation study of *in vitro* plant culture. 

To improve the production of secondary metabolites of *in vitro* plant cultures, several strategies such as supplementation and elicitation have been employed [6], for instance, the use of yeast extract [7] and jasmonic acid [8]. However, there has been no report on the use of these elicitors in *in vitro* sprout culture of *Orthosiphon aristatus* to enhance RA production. 

Fermentation, as a biotechnological process, is considered one of the oldest technologies, which is simple, natural and valuable. It can maintain and improve the safety, nutritional, sensory and shelf-life properties of many food products including plant food products [9]. Lactic acid bacteria (**LAB**) are the main bacteria responsible for the fermentation of plant food products. Most plant products can be lactic-acid-fermented, yet cucumber, cabbage and olives are the only plant products that are fermented in large volumes for human consumption [10]. 

It has been generally known that plants produce secondary metabolites that are of importance in the healthcare, food, flavor and cosmetics industries. Many biochemical changes occur during fermentation, leading to alteration in the composition of plant secondary metabolites which affects product properties such as antioxidant activity [11]. Phenolics are among the secondary metabolites of many plants that have been studied including the changes subjected to the LAB fermentation process [11,12,13,14,15]. However, most of the published reports used a terrestrial plants system as a media for studying the effect of LAB fermentation on their antioxidant properties [16,17,18,19,20,21,22]. We have not yet come across any published report describing the effect of LAB fermentation on antioxidant properties using an *in vitro* plant system. The advantage of using terrestrial plants in an LAB fermentation study is that the plant is ready to use for fermentation. Conversely, the disadvantage of using a terrestrial plant in the raw material form is that the effect of the available natural microorganisms in the plant is unavoidable. Therefore, IOSC was established to facilitate this LAB fermentation study. 

There are two main objectives for using IOSC as a model in this study, namely: (1) to find the high RA line of IOSC for LAB fermentation, and (2) to study the effect of LAB fermentation on RA content and antioxidant properties of selected IOSCs. These two objectives result in several target approaches as follows: (**a**) to select the high RA line and high antioxidant properties of IOSC by elicitation; (**b**) to characterize the antioxidant properties of IOSC using three different solvents after elicitation treatment; (**c**) to characterize the effect of LAB fermentation on the antioxidant properties of selected IOSCs; and (**d**) to identify the individual phenolic acids of IOSCs and their quantification changing with elicitation and LAB fermentation. 

## 2. Experimental Section

**Figure 1:**
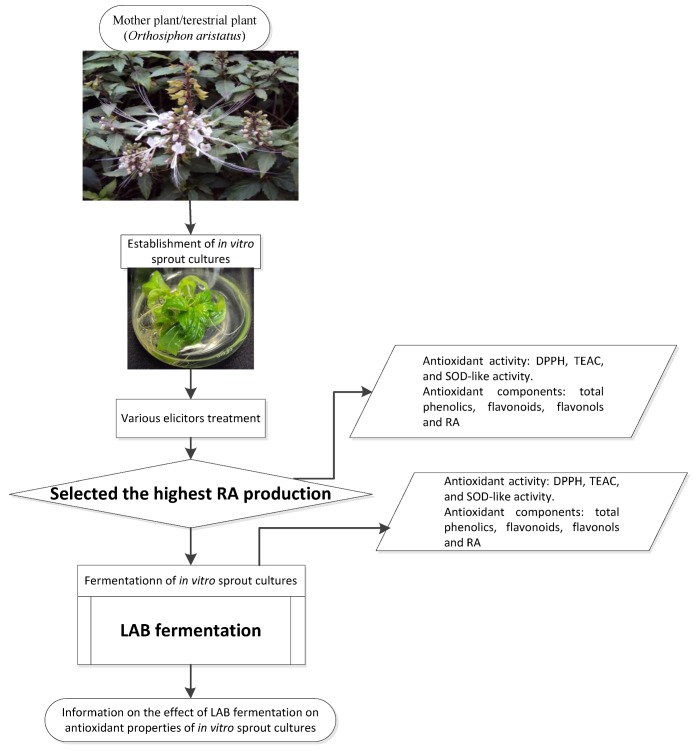
Flowchart of the effect of LAB fermentation on antioxidant properties of IOSC.

The flowchart of the current study is illustrated in Figure 1. 

### 2.1. Materials

#### 2.1.1. Plant Materials

*Orthosiphon aristatus *with article number 600646 was purchased online from Pflanzenversand Hans-Günter Röpke (refer to: http://www.pflanzenkindergarten.de). This was used to establish the *in vitro* sprout culture. 

#### 2.1.2. Culture Media

Agar Cell culture media and MS medium (Merck, Darmstadt, Germany), Oxoid Yeast Extract (Code L21) (Unipath LTD, Hampshire, England), (±) Jasmonic acid (Product of Israel, Plant cell culture tested, C_12_H_18_O_3_) (Sigma-Aldrich Chemie GmbH, Steinheim, Germany), and other materials were analytical and plant cell culture tested grades.

#### 2.1.3. Chemicals for Analysis

Gallic acid, ABTS (2,2*'*-azino-bis (3-ethylbenzthiazoline-6-sulphonic acid)) and standards of phenolic acids were obtained from Sigma-Aldrich Chemie GmbH (Steinheim, Germany); Quercetin Dihydrate (C_15_H_10_O_7_·2H_2_O ~99% HPLC) was from Fluka, Biochemika (Neu-Ulm, Germany). All used chemicals were analytical HPLC grade.

### 2.2. Methods

#### 2.2.1. Establishment of IOSC

The established procedure of IOSC was based on the previous report of Shevchenko *et al*. [5] with liquid MS medium modification as follows: MS medium (**control**), MS with jasmonic acid (100 mM) (**JA)**, and MS medium with yeast extract (5 g/L) (**YE**). 

#### 2.2.2. Fermentation Study of Selected IOSCs

The highest RA content in IOSC samples was chosen and subjected to fermentation study.

#### 2.2.3. LAB Fermentation of IOSC

*Lactobacillus plantarum ATCC 8014 *(supplied by the Department of Food Biotechnology and Food Process Engineering, Institute of Food Technology and Food Chemistry, TU Berlin, Germany) and *Lactobacillus acidophilus *NCFM, Danisco (a donation from Institute of Biotechnologie, TU Berlin) were used in this LAB fermentation study of IOSC.

#### 2.2.4. Inoculums Preparation

For preparation of inoculums, cells from stock culture were transferred to 25 mL sterile de Man, Rogosa and Sharpe (**MRS**) broth and incubated for 24 h at 37 °C*.* The growth density was measured at a wavelength of 620 nm using a spectrophotometer (PerkinElmer Precisely Lambda 25 UV/VIS Spectrometer, PerkinElmer, MA, USA). The bacteria were kept under stationary conditions until they reached an absorbance of 5.0. One ml of inoculums contained around 10^9^ colony forming units (cfu). Dilutions were made with MRS broth to bring the lactobacillus to an optical density at 620 nm of 0.1 ± 0.02 corresponding to a cell density of 10^5^–10^7^ cfu/mL.

#### 2.2.5. The Effect of IOSC Extract on Viable Cell LAB Strains Growth

The selected HPLC IOSC extract (10 mg/mL) (HPLC extraction explained below) with the highest concentration of RA was further evaluated for its inhibitory effect on the growth of LAB strains. The method of Alberto *et al*. [23] was used with modification as follows: 15% (0.75 mL) of IOSC extract was aseptically filtered with a 0.2 µm sterile syringe (VWR International Gmbh, Langenfeld, Germany) and added to 4.25 mL inoculated LAB in MRS broth (OD_620_ 0.1) in tubes and the total concentration of extract was 1.5 mg/mL. The samples tubes were incubated as explained above. The LAB growth was evaluated by direct measurement of OD_620_ every 4 h for a period of 24 h comparing the growth density with those obtained from inoculated LAB controls (OD_620_ 0.1) without IOSC extract.

#### 2.2.6. Solid State Fermentations (SSF)

This was carried out using 10 g of three-week freshly harvested IOSC inoculated with 5% (v/w) of the above culture and aseptically placed in Petri dishes in a climatic incubator controlled at 37 °C and 90% relative humidity for 24, 48 and 72 h. After fermentation, the samples were dipped in sterile water and then immersed in liquid nitrogen and freeze-dried. SSF were performed in duplicate.

#### 2.2.7. Total LAB

Viable cells of LAB were quantified at the end of fermentation (24, 48 and 72 h) using the plate enumeration method [24]. A total of 1 g of SSF was blended in 9 mL of Ringer’s solution (No. 15525, Merck, Darmstadt, Germany) and serially diluted for plate counting on MRS agar. The viable cell numbers were determined after 48 h of incubation at 37 °C under anaerobic condition produced by anaerobic kits (Anaerocult^®^ A, Merck, Darmstadt, Germany).

#### 2.2.8. Liquid State Fermentations (LSF)

LSF were performed under aseptic conditions for 24, 48 and 72 h at a temperature of 37 °C in a 1 L DCU system with digital measurement and control system for fermenter (B. Braun Biotech International, Melsungen, Germany) by aseptically placing 7.5 g IOSC (1.5% g/v) in MRS broth (500 mL) with adjusted initial OD_620_ of 0.1. Anaerobic fermentation experiments were conducted without aeration at an agitation speed of 150 rpm. LSF samples were cleaned with sterile water and dipped in liquid nitrogen and then freeze-dried.

#### 2.2.9. Viable Cells LAB Growth

LSF were started at OD_620_ 0.1 and by the end of fermentation the growth of the cells was recorded by taking OD_620_ measurements against the control without IOSC material. The growth density (OD_620_) determination was done in duplicate.

#### 2.2.10. Sample Preparation and Extraction of the IOSC

After two weeks the IOSC and LAB fermented samples were harvested, weighed and immersed in liquid nitrogen to prevent volatilization of phenolic compounds and then were freeze-dried. The lyophilized samples were ground with a flint mill (Retsch GmBH, Haan, Germany) (20,000 rpm, 2 min) to a fine powder. The dried powder of IOSC was extracted using three different solvents: 70% methanol, 80% ethanol and distilled water. The dried powders of IOSC samples were weighed accurately at 100 mg. They were treated with 5 mL of each solvent then placed in an ultrasonic water bath at 75 °C (Bandelin Sonorex Digital 10 P, Bandelin electronic GmbH & Co. KG, Berlin, Germany) for 2 h. The mixtures were centrifuged at 13,000 rpm for 5 min and the supernatants were collected as different samples. The extraction was repeated twice until the final concentration of the IOSC extract was 10 mg/mL. The extract was stored at −20 °C until further use. All samples and determinations were prepared and measured in triplicate.

#### 2.2.11. Antioxidant Properties of the IOSC

The antioxidant properties consist of characterizing the antioxidant activity and components by rapid reliable spectrophotometric measurement. The characterization of antioxidant activity was estimated based on DPPH, TEAC methods, and SOD-like activity followed by measurements of the antioxidant components: total phenolics, flavonoids, flavonols and RA. The DPPH free radical scavenging activity of the sample extract was conducted as described by Mohdaly *et al*. [25]. Then the percentage inhibition activity as relative antioxidant activity was calculated based on the following formula:


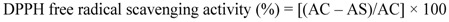


where AC is the absorbance of the control, and AS is the absorbance of the samples.

Meanwhile, the TEAC assay was performed by following standard operation procedures (SOP) of Re *et al.* [26]. A Trolox^®^ standard calibration curve of OD_734nm_ *versus* concentration of Trolox in mmol·L^−1^ was constructed. The final results were expressed as mM/g DW Trolox Equivalent (**TE**). The antioxidant activity of the freeze-dried colorants was calculated using the following equation:


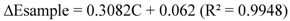


where ∆E sample is the difference in absorbance of the samples through inhibition/decolorization of the ABTS**^●^**^+^ radical scavenging activity and C is the concentration (mM/g·DW·TE).

SOD-like activity was measured according to the previously described method by Debnath *et al*. [27]. The percentage of SOD-like antioxidant activity was calculated as follow:


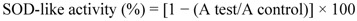


The total phenolic content of the IOSC extract was determined using the Folin-Ciocalteu method described by Mohdaly *et al.* [25]. Gallic acid was used as the standard for the calibration curve. Total phenolic content was expressed as gallic acid equivalent (**GAE**), calculated by plotting against the standard calibration curve using the following linear equation:


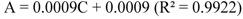


where A is the absorbance and C is the concentration (mg·GAE/g·DW).

The total flavonoids content of the IOSC extract was determined using methods previously described by Mohdaly *et al*. [25]. Freshly prepared quercetin was used as standard. The total flavonoids content was then further expressed as mg quercetin equivalent (**QE**) per gram dry weight (mg·QE/g·DW) and was calculated according to the following linear equation based on the calibration curve:


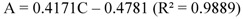


where A is the absorbance and C is the concentration (mg QE/g DW).

The concentration of total flavonols was measured according to the method of Mohdaly *et al*. [25]. The total flavonols were calculated based on the regression equation between quercetin standard and absorbance:


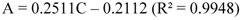


where A is the absorbance and C is the concentration (mg·QE/g·DW).

Quantification of the RA content was calculated based on a spectrophotometric method described by Kiong *et al*. [28].

#### 2.2.12. HPLC Phenolic acids in IOSC Extract

Powdered samples were weighed (20 mg accurately) and extracted within 15 min using 750 µL 70% methanol (v/v, pH 4.0, phosphoric acid) in an ultrasonic water bath with ice. The samples were centrifuged for 5 min at 6,000 rpm. The supernatants were collected and the pellets were re-extracted twice with 500 µL 70% methanol. As internal standard 40 µL of 1 mM cinnamic acid was added to the first extraction step (the recovery rate of cinnamic acid was above 90%). The combined supernatants from each sample were kept in a rotary evaporator (SPD 111V Speed Vac. Concentrator, Thermo Scientific, USA; CVC 3,000V, Vacuubrand GmbH, Wertheim, Germany) at 25 °C under vacuum to remove the solvent completely. The residues were re-dissolved in 1 mL MilliQ water (HPLC grade). The samples were filtered using a 0.22 µm filter (SPIN-X centrifuge tube filter) and then analyzed with HPLC.

The separation of phenolic compounds was performed by HPLC (UltiMate SR-3000, Dionex, Idstein, Germany), equipped with LPG-3400SD pump, WPS-3000SL automated sample injector, AcclaimPA C16-column (3 lm, 2.1 9 150 mm, Dionex) and DAD-3000 diode array detector (Dionex) and software Chromeleon 6.8. The column was operated at a temperature of 35 °C. The mobile phase consisted of 0.1% (v/v) phosphoric acid in water (eluent A) and of 40% (v/v) acetonitrile (eluent B). A multistep gradient was used for all separations with an initial injection volume of 40 µL and a flow rate of 0.4 mL·min^−1^. The multistep gradient was used as follows: 0–1 min: 0.5% (v/v) B; 1–10 min: 0.5–40% B; 10–12 min: 40% B; 12–18 min: 40–80% B; 18–20 min: 80% B; 20–24 min: 80–99% B; 24–30 min: 99–100% B; 30–34 min: 100–0.5% B; 34–39 min: 0.5% B. Simultaneous monitoring was performed at 290, 330 and 254 nm. Diode array detection was used for the identification of the compounds. Retention times and UV/visible absorption spectra of the peaks were compared with those of the authentic standards. 

### 2.3. Statistical Analysis

Experimental results in this study are reported as means ± standard deviation (S.D.) of five replicates. The data were subjected to analysis of variance (ANOVA) and post-hoc Tukey’s Honestly Significant Different (**HSD**) tests. The level of statistical significance was set at p-values <0.05 using SPSS version 16.0 (SPSS Inc. Chicago, IL, USA). Correlation analyses between the parameters were carried out using the “ProcCorr” procedure in SAS (2002-10) for Windows, version 9.3 (SAS Institute, Cary, NC, USA).

## 3. Results and Discussion

### 3.1. Selection of High RA Line and Antioxidant Properties of IOSCs by Elicitation for the LAB Fermentation Study

The main difference between *in vitro* sprout culture and *in vitro* tissue culture of *O. aristatus* is the aggregate state of the nutrient medium. The nutrient medium of the sprout culture, regardless of nutrient content and sucrose concentration on it, is always liquid. Another difference in sprout culture is that it is always agitated. Further information about *in vitro* sprout culture was explained by Sevchenko *et al.* [5]. Preliminary research showed that *Orthosiphon aristatus* in *in vitro* sprout culture was capable of developing faster and had higher biomass compared to that of *in vitro* tissue culture which is important in producing tea leaves. Data of two-week IOSC behavior subjected to elicitation are depicted in Table 1. 

**Table 1:** Data of two-week IOSC behavior subjected to elicitation.

Sample group	IOSC Length	Number of leaves	Number of roots	Fresh weight (FW) (g)
Control	5.02 ± 0.58	9.40 ± 2.88	4.40 ± 1.14	6.29 ± 1.44
JA	3.88 ± 0.94	5.80 ± 1.30	3.80 ± 2.17	4.23 ± 0.86
YE	3.10 ± 0.80	5.20 ± 1.30	3.60 ± 1.95	5.40 ± 1.18

Data reported as average ± SD with n = 5.

Control = MS medium; JA = MS medium with jasmonic acid; YE = MS medium with yeast extract.

#### 3.1.1. Antioxidant Activity of IOSC after Elicitation

**Figure 2:**
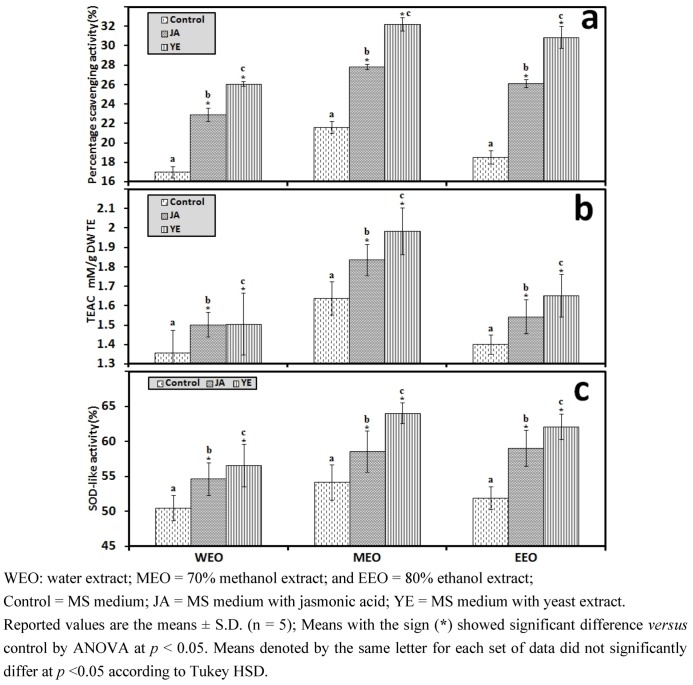
Antioxidant activity of the IOSC subjected to elicitation using three different solvents (**a**) DPPH scavenging activity; (**b**) TEAC antioxidant activity; and (**c**) SOD-like activity.

In response to elicitation treatment, an increase in the percentage of antioxidant activity was noticed for all samples compared to the control (Figure 2). Yeast extract elicitation resulted in the highest percentage of DPPH scavenging activity (32.21 ± 0.69% for the 70% methanol extract as seen in Figure 2(a). Similarly, the highest TEAC antioxidant activity was recorded for IOSC samples induced with yeast extract elicitor and corresponded to 1.51; 1.99; and 1.65 mM/g TE for the IOSC; WEO, MEO and EEO respectively (Figure 2(b)). Furthermore, in terms of SOD-like activity attained (Figure 2(c)), elicitation showed a significant increase in the percentage of SOD-like antioxidant activity. Yeast extract elicited IOSC with 70% methanol as solvent had the highest SOD-like activity among all treatments. 

#### 3.1.2. Antioxidant Components of IOSC after Elicitation

**Figure 3:**
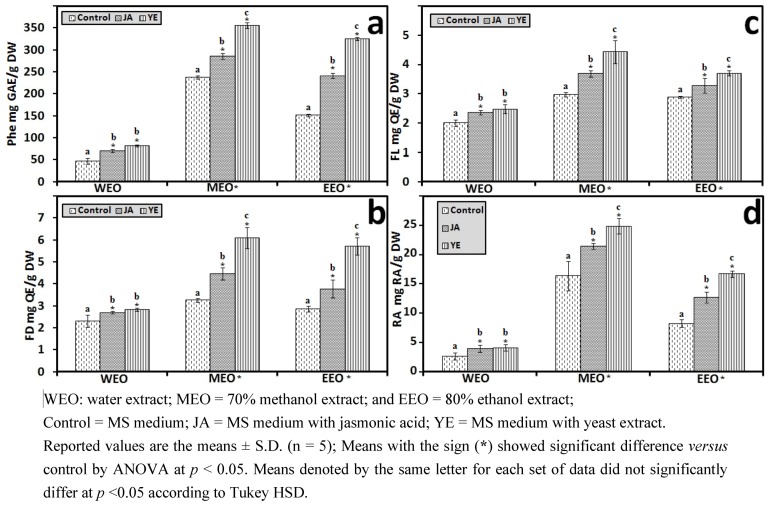
Antioxidant components of the IOSC subjected to elicitation using three different solvents for extraction (**a**) total phenolics (**Phe**); (**b**) flavonoids (**FD**); (**c**). flavonols (**FL**); (**d**) RA.

In accordance with the results of antioxidant activity, the total phenolics, flavonoids, flavonols, and RA concentration responded positively to elicitation with increases in content, suggesting that the elicitors stimulated the synthesis of these secondary metabolites (Figure 3). Among all the samples, yeast extract IOSC had the highest amount of total phenolic compounds, flavonoids, flavonols, and RA content for all three tested solvents. The total phenolics from the ethanol extraction of IOSC with yeast extract used as elicitation were two fold (324.56 ± 3.73 mg·GAE/g·DW) compared to the IOSC control (151.22 ± 2.41 mg GAE/g DW) (Figure 3(a)). A similar pattern was also observed for total flavonoids, flavonols, and RA level from IOSC with yeast extract elicitation, which had the highest amount (Figure 3(b–d)). The total amount of flavonoids was 2.96; 4.67 and 3.95 mg·QE/g·DW and total flavonols was 2.47; 3.85 and 3.42 mg·QE/g·DW for WEO, MEO and EEO respectively as illustrated in Figure 3(c,d). These results indicated that the increase in antioxidant activity was in conjunction with increases in antioxidant components in the elicited samples.

#### 3.1.3. Solvent for Extraction

In response to the different solvents used for extraction, 70% methanol exhibited the highest yield of total phenolics, flavonoids, flavonols and RA. It consequently resulted in the strongest antioxidant activity. This finding corresponded with previous research on plant extraction carried out by Lee *et al.* [29] and Sun *et al*. [13], where they investigated the influence of different solvents on extraction. They found that the methanol extract showed the highest yield of phenolic compounds and the strongest antioxidant activity. A similar result was also reported by Azlim Almey *et al*. [30] confirming methanol as the most efficient solvent for the extraction of phenolic compounds in comparison to ethanol and water. They further explained that the highest yield of phenolics in the methanol extract was due to the ability of methanol to inhibit polyphenol oxidase (**PPO**) that causes oxidation of phenolics and lessens its evaporation compared to ethanol and water.

#### 3.1.4. HPLC Phenolic Acids of IOSC after Elicitation

**Figure 4:**
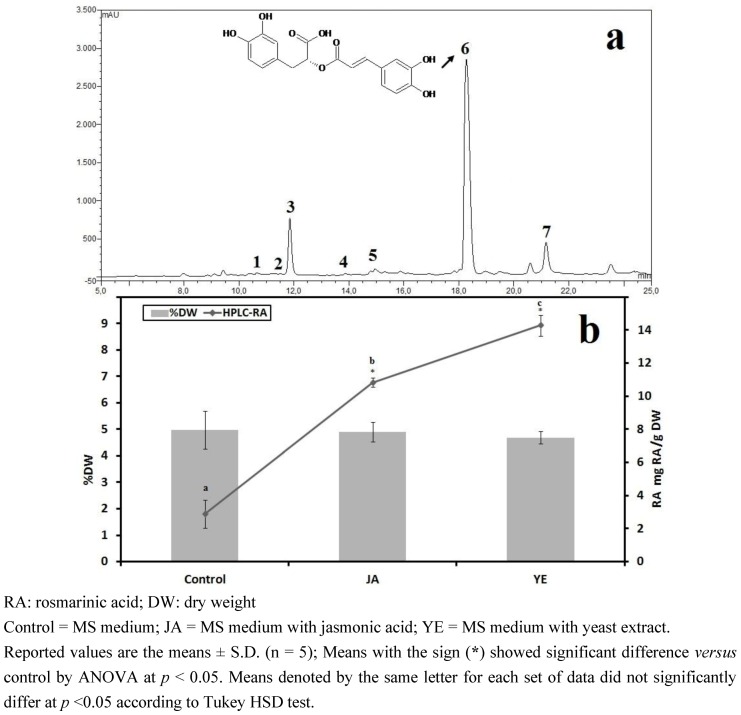
(**a**) Typical HPLC chromatogram of IOSC extract subjected to elicitation: 1: vanillic acid; 2: chlorogenic acid; 3: caffeic acid; 4: p-coumaric acid; 5: sinapic acid; 6: rosmarinic acid; and 7: cinnamic acid as internal standard (peak identification based on standard compounds); and (**b**) HPLC-RA quantification of IOSC after elicitation.

From the HPLC analysis of phenolic acids as shown in Figure 4(a), the elicitation confirmed a significant increase in RA content compared to the control. The basal level of RA content in the control sample was 2.89 ± 0.86 mg/g·DW (Figure 4(b)), meanwhile in IOSC samples elicited with JA it was 3.75-fold higher (10.83 ± 0.27 mg/g·DW). Nevertheless, the highest elicitation effect was seen in IOSC samples treated with yeast extract resulting in 4.95-fold RA content (14.28 ± 0.64 mg/g·DW) in comparison to that of the control. These results showed that jasmonic acid and the yeast extract of the elicitors were very effective in inducing the production of RA in IOSC. These results were consistent with those previously described by Ogata *et al*. [31] who found that the addition of yeast extract and methyl-jasmonate rapidly enhanced the accumulation of RA in the culture of *Lithosermum erythrorhizon*.

Moreover, Kim *et al*. [32] reported that the stimulation of RA biosynthesis in *Agastache rugosa O. Kuntze* was very successful in response to the addition of yeast extract which could raise RA content up to 5.7-fold compared to the non-elicited suspension cells. Previous reports suggested that the increase of RA content by yeast extract treatment coincided with the increase in phenylalanine ammonia-lyase (PAL) activity, indicating the important role of PAL activity in the regulation of RA biosynthesis [7,33]. The increase of the level of RA can be explained by the fact that IOSCs, like other plants, carry out an elicitor-mediated defense response by inducing phenylpropanoid pathways resulting in production of more RA. As a result, yeast extract elicitor was found to be the best strategy for RA production in comparison to jasmonic acid as equally reported in other studies [7,33]. Therefore, IOSC with yeast extract elicitor was used to study the effect of LAB fermentation in IOSC.

In addition, the responses of other phenolic acids are provided in Table 2. Yeast extract elicitation, however, caused an increased in vanillic, caffeic acid and *p*-coumaric acid by 3.72, 3.46 and 3.50 fold of the control, respectively. In other plant species, *Mallus domestica* cell culture, p-coumaric acid and chlorogenic acid were more responsive to the yeast extract elicitation and enhanced more dramatically with 5.1 fold and 2.7 fold of that of the control level [6]. These observations indicated that yeast extract elicitation also enhanced the production of other phenolic acids and strengthened the choice of yeast extract-IOSC for the LAB fermentation study.

**Table 2:** Individual response of phenolic acids subjected to elicitation.

Other phenolic acids	Control(mg/g·DW)	Fold of control	
		JA	YE
Vanillic acid	2.28 ± 0.27	2.80	3.72
Chlorogenic acid	0.49 ± 0.07	1.10	1.90
Caffeic acid	4.62 ± 0.15	1.30	3.46
p-Coumaric acid	0.27 ± 0.03	2.40	3.50
Sinapic acid	1.12 ± 0.04	2.5	1.82

Control = MS medium; JA = MS medium with jasmonic acid; YE = MS medium with yeast extract.

### 3.2. LAB Fermentation Study of Selected IOSC

To the best of our knowledge, this is one of the first studies using *in vitro* plants as a media for LAB fermentation focusing on rosmarinic acid and related antioxidant properties. 

#### 3.2.1. The Effects of IOSC Extract on Viable Cell LAB Growth

There are several methods to determine LAB growth rates. Plate count is by far the most reliable method to establish growth curve, but it is extremely laborious. An alternative is the measurement of optical density (**OD**). OD measurement is adequate for modeling the growth of food-fermenting organisms, where the emphasis is exclusively on conditions that allow growth to a high population density. However, it is not reliable enough when it is use to assess the growth of food pathogens or spoilage organisms [34,35]. Therefore, in this fermentation study, the antibacterial effect of selected extracts on the growth of LAB strains was further evaluated by determining their growth density OD_620_.

**Figure 5:**
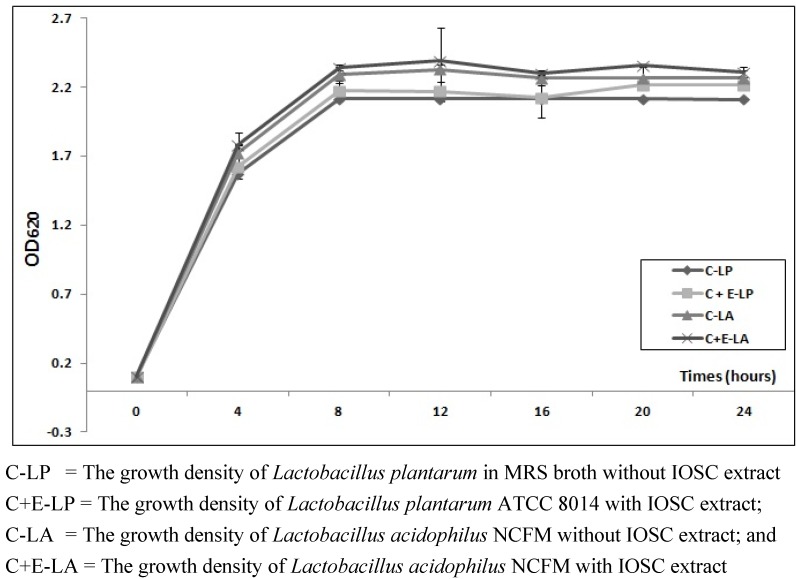
Growth density of *Lactobacillus plantarum* ATCC 8014 and *Lactobacillus acidophilus *NCFM with/without IOSC extract.

In general, based on absorption value increase measured at 620 nm, the added IOSC extract resulted in insignificant difference (p > 0.05) compared to the growth density of the pure culture (Figure 5). These results mean that the IOSC extract at the concentration 1.5 mg/mL used had no inhibitory effect on the growth of LAB strains used in this present study. The only report found on the *O. aristatus* terrestrial plant showed that the aqueous extract of *O. Aristatus* demonstrated antibacterial activity against two serotypes of *Streptococcus mutans* with minimal inhibition concentration (MIC) of 7.8–23.4 mg/mL [36] which was much higher than 1.5 mg/mL. These results were in accordance with the previous investigation by Puupponen-Pimiä *et al*. [37] who studied the antimicrobial effect of the extract of several berries using 70% acetone as solvent on the growth of LAB strains (including *L. plantarum*). They reported that the berry extract at low concentration of 1 mg/mL had no antimicrobial effect on the LAB strains used. A similar finding was recently reported by He *et al.* [21] that the *Codonopsis lanceolata* water extract (0.25 g/mL) provided a good substrate for LAB growth. 

#### 3.2.2. Viable Cell Growth of SSF and LSF

The growth of LAB in line with the production of organic acids as well as other possible antimicrobial substances restricted the growth of aerobic heterotrophic bacteria [11]. The growth of LAB in SSF samples remained unchanged for 24 h samples for both **LP** and **LA** inoculated SSF. The total viable count of inoculated LAB strains OD_620_ 0.1 was 3.2 × 10^5^ cfu/g for *L. plantarum* and 5.8 × 10^5^ cfu/g for *L. acidophilus *and after 24 h fermentation for SSF samples, the viable cells of LAB were 3.2 and 8.1 × 10^5^ cfu/g for SSF-LA and LP, respectively (Table 3(a)). A one log decrease to 5.2 × 10^4^ cfu/g was observed after SSF treatment with LP for 48 h. A similar trend was observed with SSF LA-48. By the end of fermentation, the total viable cells remained unchanged at 10^4^ cfu/g for both SSF samples.

Meanwhile, the changing of the viable cell of LAB strains in LSF after 24, 48 and 72 h was evaluated by OD_620_ determination. In general, the addition of sterile IOSC (1.5% g/v) in growth density of LAB strains (*L. plantarum* and *L. acidophilus*) in a DCU fermenter resulted in an insignificant difference compared to the control without IOSC (Table 3(b)).

**Table 3(a):** Total LAB of SSF.

Strains	Time (h)	Total LAB *
*L. plantarum*	Inoculated OD_620_ 0.1 = 3.2 × 10^5^ cfu/g	
	24	8.1 × 10^5^ cfu/g
	48	5.2 × 10^4^ cfu/g
	72	2.5 × 10^4^ cfu/g
*L. acidophilus*	Inoculated OD_620_ 0.1 = 5.8 × 10^5^ cfu/g	
	24	3.2 × 10^5^ cfu/g
	48	1.8 × 10^4^ cfu/g
	72	3.0 × 10^4^ cfu/g

* Values are results of at least two independent experiments.

**Table 3(b):** The growth density OD_620_ of LSF.

MRS broths	Time (h)	OD_620_
*L. plantarum* (without IOSC; control)	24	2.221 ± 0.010
	48	1.661 ± 0.009
	72	1.777 ± 0.013
*L. plantarum* with IOSC	24	2.225 ± 0.021
	48	1.664 ± 0.011
	72	1.871 ± 0.011
*L. acidophilus *(without IOSC; control)	24	2.151 ± 0.008
	48	1.558 ± 0.015
	72	1.831 ± 0.007
*L. acidophilus* with IOSC	24	2.226 ± 0.009
	48	1.560 ± 0.012
	72	1.839 ± 0.014

#### 3.2.3. Solid State Fermentation (SSF) of IOSC

**Figure 6:**
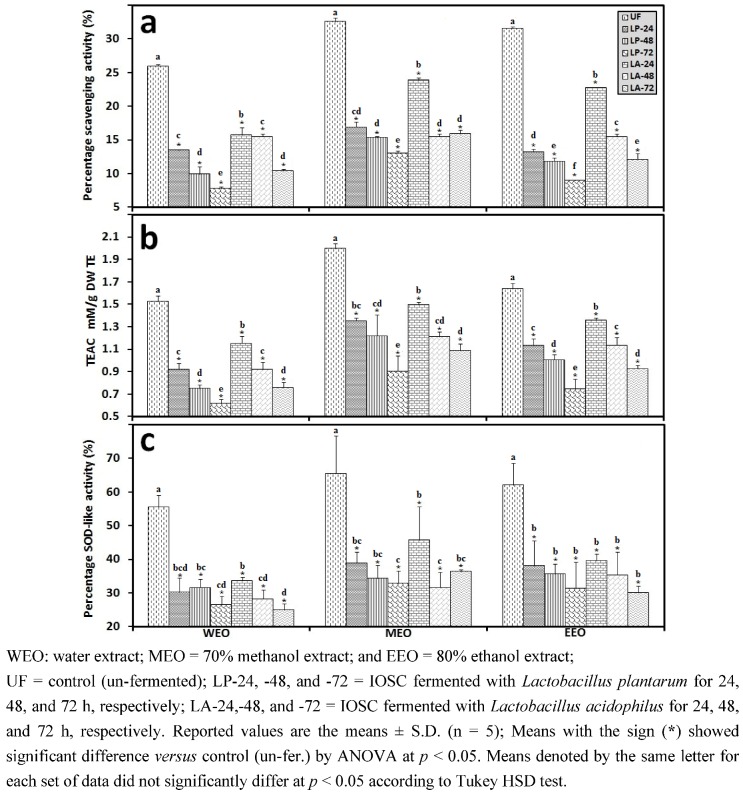
Antioxidant activity of IOSC subjected to SSF using three different solvent**s **(**a**) DPPH scavenging activity; (**b**) TEAC antioxidant activity; and (**c**) SOD-like activity.

Figure 6(a) illustrates the percentage DPPH scavenging activity of SSF-IOSC (unfermented (control) and fermented) using three different solvents. As is shown, SSF significantly influences the DPPH radical scavenging activity of IOSC. Unlike most of the latest published reports on LAB plant fermentation studies [16,1,2,3,4,5,6,7,8,9,10,11,12,13,14,15,16,17,18,19,20,21] which resulted in an increase in antioxidant activity, SSF with LAB caused a weakening percentage of DPPH radical scavenging activity of IOSC compared to the control for all three solvents tested. The longer period for SSF caused more losses in the DPPH radical scavenging activity. The percentage for 30 min tested DPPH radical scavenging activity of UF water extract was 25.98 ± 0.23% which decreased considerablyy to the level of 10.41 ± 0.21% after 72 h SSF treatment inoculated with LA. A higher decrease was observed in SSF with LP inoculation and the remaining percentage of DPPH radical scavenging activity was only 7.79 ± 0.23% after a 72 h period of fermentation.

In comparison to the DPPH free radical scavenging method, the TEAC antioxidant activity method was also applied. A similar trend was observed (Figure 6(b)), a significant reduction (*p* < 0.05) of TEAC antioxidant activity was noted as a response to SSF treatment either LP or LA inoculation (Figure 6(b)). The rate of TEAC value reduction after 72 h SSF fermentation with LA is as follows: 50.28 ± 1.56%, 45.59 ± 2.02% and 43.47 ± 0.37% for water, 70% methanol and 80% ethanol extract, respectively. A higher reduction rate of the TEAC value was obtained in SSF for 72 h with LP inoculation, 59.63 ± 3.00%, 54.65 ± 7.83% and 54.63 ± 4.08% for water, 70% methanol and 80% ethanol extract, respectively.

In terms of the SOD-like activity results attained, LAB fermentation caused significant depletion (p < 0.05) of its activity (Figure 6(c)). Similarly, the diminishing result of antioxidant activity using SOD-like methods in *Vigna sinensis* var. carilla due to natural fermentation was also reported by Doblado *et al*. [38] and this decreasing trend of SOD-like activity was confirmed with our result.

**Figure 7:**
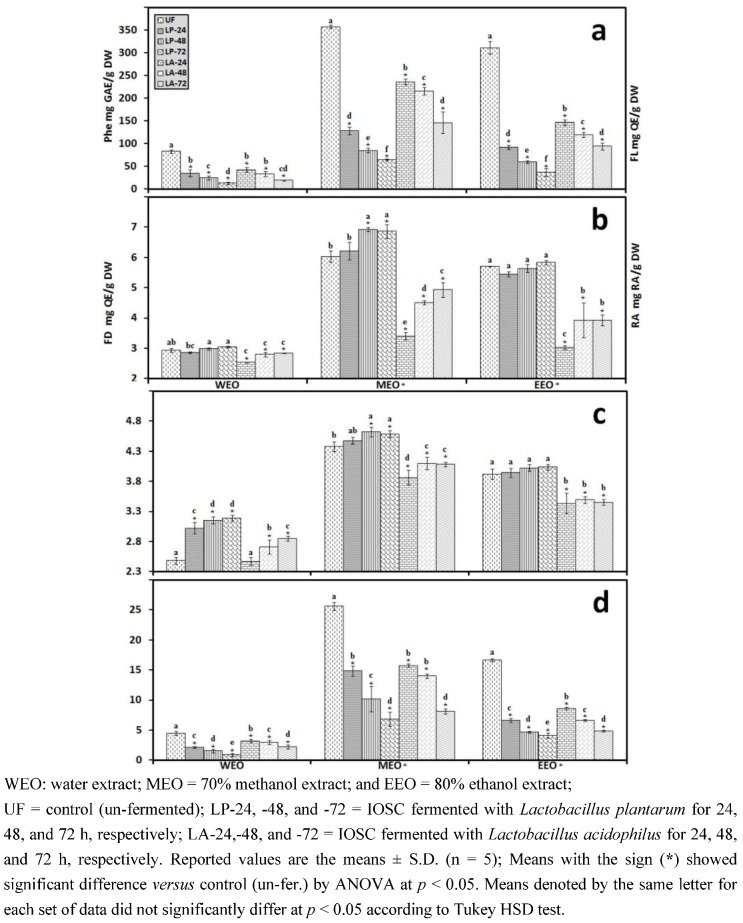
Antioxidant components of IOSC subjected to SSF using three different solvents for extraction (**a**) total phenolics (**Phe**); (**b**) flavonoids (**FD**); (**c**) flavonols (**FL**); (**d**) RA.

The losses of antioxidant activity of LAB fermented SSF can be linked to the sharp decline in the total phenolics and RA during SSF. The data are presented in Figure 7. 

The knowledge that phenolic degradation is mainly caused by polyphenol oxidase (PPO) is well known. This phenomenon clearly indicated that the degradation of phenolic compounds occurred during the SSF process by an enzymatic process involving PPO. The obtained results were in agreement with a previous study on *L. plantarum* inoculation of potherb mustard (*Brassica juncea* Coss.) pickling fermentation by Fang *et al.* [39] that resulted in a decrease in antioxidant activity associated with the reduction of total phenolics during fermentation. Here it was further explained that the decrease of total phenolics indicated that the phenolics degradation by enzymatic and non-enzymatic processes occurred during fermentation. A similar declining antioxidant properties trend was also reported on the fermentation study of Chetoui olives (black, green and varicolored olive) using *L. plantarum* with spontaneous fermentation by Othman *et al.* [40]. They reported that the antioxidant activity of Chetoui olives after *L. plantarum* and spontaneous fermentation decreased by 50–72%. In our study, the drastic decrease of antioxidant activity was connected with an important loss of total phenolics and rosmarinic acid.

Figure 7(b) shows the effect of LAB SSF on total flavonoids and flavonols of IOSC. The flavonoid and flavonol content in response to *L. plantarum* SSF showed a modest decrease after 24 h fermentation for all solvents used for extraction. A higher rate of depletion in total flavonoids and flavonols occurred after 24 h of *L. acidophilus* SSF. Interestingly, both LAB SSFs gradually increased for longer fermentation (48 and 72 h). An increase in total flavonoids including flavonols during plant fermentation has been reported by many researchers [13,17,18,20,41,42,43]. Cho *et al.* [44] showed that the increase in flavonols in fermented Soybean by *Bacillus pumilus* HY1 was the result of the esterase and tannase activities of LAB during fermentation. This phenomenon indicated a possible conversion of fermentation-stimulated metabolites. These results suggested that some flavonoids could be degraded during fermentation and/or may be produced from the degradation of phenolic compounds [45]. 

In response to the different solvents used for extraction, similar findings were in agreement with the elicitation result treatment. Results showed that 70% methanol, regardless of LAB fermentation, produced the highest yield of total phenolics, flavonoids, flavonols and RA and consequently exhibited the strongest antioxidant activity (DPPH, TEAC and SOD-like activity). This finding corresponded with previous plant fermentation studies by Lee *et al*. [29] and Sun *et al*. [13] employing different solvents for extractions of which the methanol extract showed the highest yield of phenolic compounds as well as the strongest antioxidant activity. 

#### 3.2.4. Liquid State Fermentation (LSF) of IOSC

**Figure 8:**
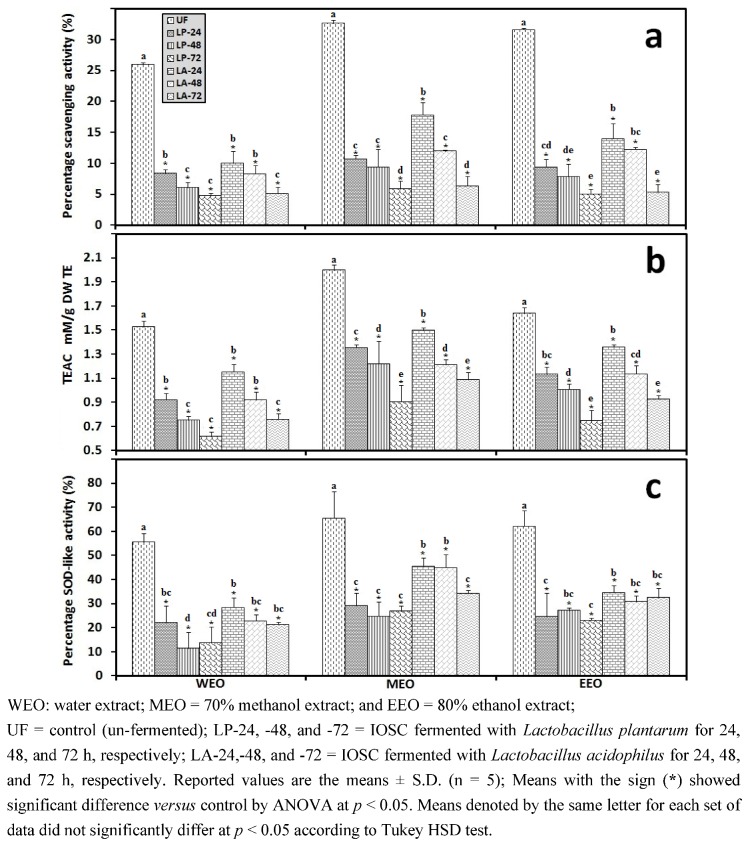
Antioxidant activity of IOSC subjected to LSF using three different solvents (**a**) DPPH scavenging activity; (**b**) TEAC antioxidant activity; and (**c**) SOD-like activity.

In contrast to the SSF method which utilized 5% of LAB starters, a further change in antioxidant properties of IOSC was observed in LSF predominated by LAB starters in MRS medium. The data are shown in Figure 8. Many studies have shown that the utilization of LAB in plant fermentations can increase antioxidant properties [16,19], however, such an effect was not observed in the LAB fermentation of IOSC as examined in this study. There was a remarkable decrease in the detectable antioxidant activity (DPPH, TEAC, and SOD-like activity Figure 8 (a,b,c respectively)) during the LSF process for 24, 48 and 72 h regardless of the starter organisms employed for fermentation. 

In general, a higher rate of reduction in antioxidant activity was observed in LSF than in SSF and as observed in SSF the *L. plantarum* fermentation caused greater losses of antioxidant activity in comparison to *L. acidophilus*. This lessening antioxidant activity of plant extracts by fermentation has been reported by several investigators [46,47,48,49]. Natural fermentation caused a reduction of TEAC value of 23% in *Lupinus albus* var. *multolupa* [47], 4% in *Cajanus cajan * [48], and 9–29% in soybean [49], while that with *A. oryzae* or *L. plantarum* in *Lupinus angustifolius cv. Zapaton* resulted in a decrease of 18% and 24% of the TEAC values, respectively [46]. Inoculated fermentation with *A. oryzae*, *R. oryzae*, or *L. plantarum* in soybean caused a decrease of TEAC by 40, 26, and 9–13%, respectively [49]. Similarly, Chang *et al.* [50] reported that the loss in the antioxidant activity was caused by the microbial activity during fermentation which was confirmed by our result.

In terms of decreasing SOD-like activity obtained due to LSF in Figure 8(c), the result was inconsistent with the report by Fernandez-Orozco *et al. *[46] on *Lupinus angustifolius* cv. *Zapaton,* fermented by using *A. oryzae*, *R. oryzae*, or *B. subtilis* where the fermentation process resulted in a drastic decrease (98–100%) of SOD-like activity. A large reduction in SOD-like activitywas also observed in fermented soybean [49]. Additionally, using a different antioxidant activity method of Peroxyl Radical-Trapping Capacity (PRTC), Doblado *et al*. [38] reported that the application of liquid fermentation on *Vigna sinensis* var. carilla caused the reduction of PRTC antioxidant activity.

**Figure 9:**
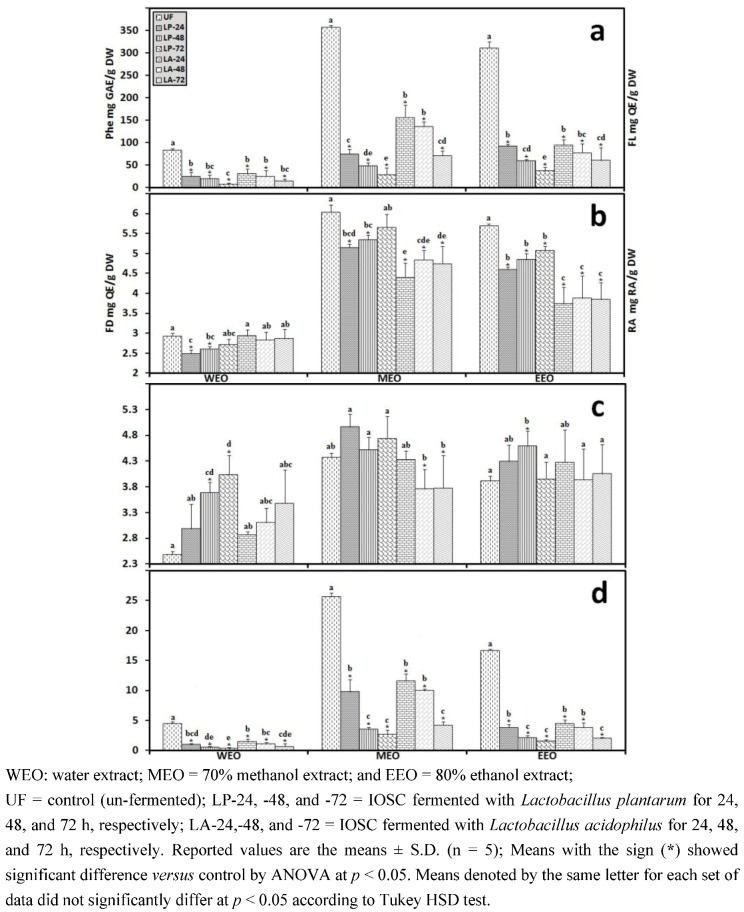
Antioxidant components of IOSC subjected to LSF using three different solvents for extraction (**a**) total phenolics (**Phe**); (**b**) flavonoids (**FD**); (**c**) flavonols (**FL**); (**d**) RA.

The gradually weakening antioxidant activity corresponded to the sharp depletion in antioxidant components (total phenolics and RA). Figure 9 shows the components of the antioxidant levels of control (unfermented) and LSF of IOSC using LAB. The total phenolics decreased considerably in both LSF inoculated with *L. acidophilus* and *L. plantarum* (Figure 9(a,b)), and a lower level of these antioxidant components was obtained in LP-LSF samples compared to LA-LSF samples. Concerning the effect of fermentation on RA level, a similar reduction trend was also found (Figure 9(d)). 

This result was in agreement with the previous report by Frias *et al.* [47] who reported a decrease in antioxidant components of *Lupinus albus var. multolupa* carried out by natural fermentation or inoculated with *L. plantarum*. *A s*imilar observation was also reported by Landete *et al.* [51] who showed that the growth of *L. plantarum *led to the depolymerization of high-molecular-weight phenolic compounds, with a resultant decolorization of the fresh olive mill wastewater and a significant reduction in total phenolics. A decrease in the total phenolics with LSF as reported here showed that phenolics were degraded and utilized by fermented microorganisms as reported by other researchers [52,53]. Moreover, in terms of decreasing flavonoids by fermentation, it has been reported that flavonoids glycosides were metabolized within a 72 h fermentation period using an *in vitro* fermentation human fecal micro-flora system [54].

Interestingly, as found in SSF, an increase in total flavonols in LSF was also observed (Figure 9(c)). Fermentation led to increased total flavonols reported by Cho *et al*. [18] and Cho *et al* [44]. They explained that the esterase activity and total flavonols increased significantly with a corresponding decrease in flavonol gallates during the fermentation [44]. 

Turning to the solvent used for extraction, a similar pattern to elicitation and SSF in that the highest antioxidant activity and components were observed in the LSF-IOSC extract with methanol, while the lowest levels were found in a water extract. The results supported and confirmed that 70% methanol was also the most suitable for extraction of antioxidant component, bringing about a higher antioxidant activity, owing to its high polarity and good solubility for both flavanoids and components of the plant materials. 

#### 3.2.5. HPLC Analysis of IOSC after LAB Fermentation

**Figure 10:**
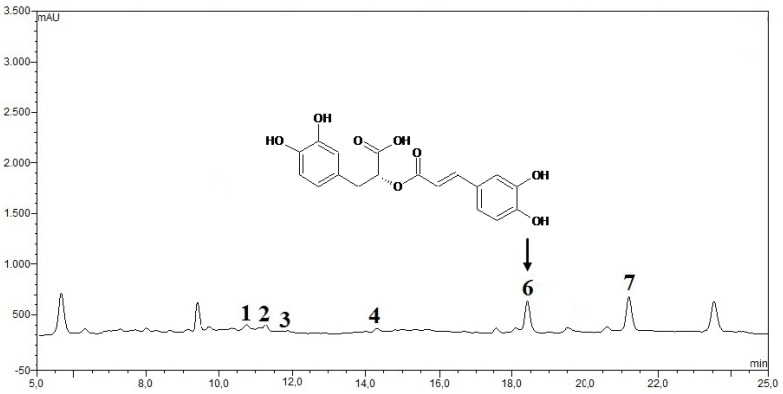
Typical HPLC chromatogram of IOSC extract subjected toLAB fermentation: 1. vanillic acid; 2. chlorogenic acid; 3. caffeic acid; 4. p-coumaric acid; 5. sinapic acid; 6. rosmarinic acid; and 7. cinnamic acid as internal standard (peak identification based on standard compounds).

Individual phenolic compounds were analyzed by HPLC to further examine the effect of LAB fermentation on IOSC and in particular RA as the compound of interest in this study. The determination of the phenolic proﬁle in plants, in particular fermented plants is difficult. The reason being that the majority of the reference compounds are not commercially available and in order to carry out quantitative analysis of phenolics in plant extracts, the hydrolysis procedure, particularly the acid hydrolysis has been used very often to simplify the analysis and thus reduce the number of derivatives [55]. In our study, hydrolysis of phenolic acids occurred by the addition of acid to methanol for extraction and the phenolic proﬁles of IOSC were determined using the HPLC-DAD method proceeded by simultaneous monitoring at 290, 330 and 254 nm. The typical HPLC phenolic acids chromatograms of IOSC extract are presented in Figure 10.

Several identified phenolic acids, vanillic, chlorogenic, caffeic, p-coumaric, sinapic and RA, were eluted in the chromatograms (Figure 10). IOSCs were found to accumulate RA as the major phenolic of all identified phenolic constituents by HPLC analysis followed by caffeic acid as the second major phenolic acid in IOSC. This finding was in agreement with the report on the analysis of this plant by Sumaryano *et al*. [56] who equally identified RA and caffeic acid as the major phenolic constituents in the methanol extract. There are several major unknown peaks predicted to be degradation products of LAB fermentation. The changing phenolic acids subjected to LAB fermentation, SSF and LSF, are depicted in Table 4.

**Table 4:** Individual response of phenolic acids to the SSF and LSF LAB fermentation of IOSC for 48 h.

Phenolic acids	Control (un-fermented)	% decrease *L. plantarum * ATCC 8014		% decrease *L. acidophilus *NCFM	
	(mg/g·DW)	SSF	LSF	SSF	LSF
Rosmarinic acid	14.28 ± 0.64	55.7	72.3	46.8	56.2
vanillic acid	8.48 ± 0.06	23.5	43.2	20.4	24.2
chlorogenic acid	0.93 ± 0.11	27.6	40.1	23.6	35.6
caffeic acid	9.06 ± 0.33	42.4	49.5	37.3	44.9
p-coumaric acid	0.95 ± 0.07	29.6	37.6	28.6	32.8
sinapic acid	2.04 ± 0.03	26.8	33.9	21.6	29.8

As observed in our HPLC results, both fermentations, SSF and LSF, caused a significant decrease of individual specific phenolic acids which has also been reported by several researchers [52,53,57,58]. Hegde, *et al*. [53] reported the drastic decrease in feruclic, coumaric and syringic acids of wheat and rice brans respectively after *A. niger* fermentation at 96 h, while gallic and protocatechuic acids were completely degraded in 96 h. Degradation of these phenolic acids clearly indicated the induction of various phenolic acid esterases. It was associated perhaps with the oxidation of those phenolic acids or even utilized by fermented microbes for various end uses [53]. Similarly, the concentration of the hydroxybenzoic acids fell slightly at the end of alcoholic fermentation while the hydroxycinnamic acids (caffeic, coumaric and ferulic acids) decreased due to dilution effects in wine fermentation [57]. Also, the content of 4-hydroxybenzoic and syringic acid decreased five- and two-fold respectively in palm oil fermented with *A. niger* [52]. The degradation and formation of phenolic acids clearly indicates the action of various enzymes especially the esterases and cell-wall degrading enzymes secreted by the fermented microorganism [52].

Moreover, the *S. Cerevisiae* fermentation of *Rubus coreanus Miq*. using different fractions; the soluble phenolic fraction (Fr. A), the soluble ester-type phenolic fraction (Fr. B) and the insoluble ester-type phenolic fraction (Fr. C), resulted in a decrease of gallic and ferulic acid in Fr. A and of salicylic, caffeic, and cinnamic acid contents in Fr. C [58]. These decreased phenolic acid contents could be attributed to either microbial oxidation, reduction, or the degradation of the phenolic compounds by the fermenting microbes [58]. In wine fermentation, it has been suggested that the decrease in the concentration of *trans*-caftaric and *trans*-*p*-coutaric acids until disappearance could be linked to lactic acid bacteria metabolism [59].

It has been generally known that *L. plantarum* has the ability to degrade many types of phenolic acids [60]. From the hydroxycinnamic group, *L. plantarum* is able to metabolize *p*-coumaric, caffeic, ferulic, and *m*-coumaric acid and from hydroxybenzoic acids both gallic and protocatechuic acid. Decarboxylation and reduction of the phenolic acid reactions were involved in their metabolism. The formation of ethyl derivatives from the degradation of the hydroxycinnamic group and from some compounds like pyrogallol and catechol, which might be beneficial for the growth and metabolism of this bacterium, were detected [60]. Another type of LAB, *L. brevis* was shown to degrade p-coumaric acid and caffeic acids via decarboxylation and their vinyl derivatives were detected [61]. However, there was no report on the metabolism of phenolic acids in *L. acidophilus*. Unfortunately, with our HPLC methods, the degradation products could not be identified. The above mentioned fermentation reactions can significantly influence food quality through the development and degradation of polyphenols. Therefore, further work on the identification of the degradation products and enzymes involved is required to improve our understanding of phenolic acid degradation by LAB.

As for the effect of LAB fermentation on RA in IOSC leaves, the HPLC analysis of phenolic acids confirmed that RA was predominant in soluble phenolics and was sharply reduced in both SSF and LSF compared to UFT (unfermented). LSF suffered a higher rate of decreasing RA content than SSF. In terms of type LAB inoculums, *L. plantarum* caused a more dramatic degradation compared to *L. acidophilus*. The relatively high temperature of 37 °C for LAB growth was predicted as contributing to the great losses of RA. This finding was in agreement with the report by Fletcher *et al*. [62] who showed that heat treatment at only 30 °C could significantly reduce the content of RA and phenolic compounds. Similarly, tea fermentation of *C. asiatica* caused a reduction in RA [63]. The RA level decreased significantly from 282 ± 42 µg/g in the unfermented to 158 ± 40 µg/g in partially fermented *C. asiatica* leaves and there was a further reduction to 58 ± 1 µg/g in the fully fermented *C. asiatica* tea leaves [63].

The fact both LAB fermentations SSF and LSF inoculated with *L. plantarum* and *L. acidophilus* using sterilized-*in vitro* sprout culture of *Orthosiphon aristatus* as model system had a lower level of phenolics acids measured by HPLC analysis, clearly supports and confirms the result of spectrophotometric measurement that fermentation not only depromotes the antioxidant activity with all mechanisms of antioxidant measurements, but also is accompanied by a notable decrease of antioxidant components. 

#### 3.2.6. Correlation Analysis between the Experimental Variables

In regard of acquiring a holistic approach to the IOSC antioxidant properties and respective of their partial chemical constituents, a Pearson correlation matrix between all these variables was performed. It has been suggested that the antioxidant activity of plant extracts is mainly due to phenolic compounds in the plant extract [25], although Azlim Almey *et al*. [30] found only a weak correlation. Additionally, antioxidant potency of the extract is not proportional to the total phenolic content, suggesting that the antioxidant activity is not simply a function of phenolic concentrations [64].

However, from our experimental results, a positive correlation was found. In response to elicitation, an increasing antioxidant activity was also followed by an increase in total amount of phenolics and RA and the reverse was true for the LAB fermentation results. It has been reported that not all antioxidant components have the same antioxidant activity, and gallic acid and rosmarinic acid were found to be the most potent antioxidants among the simple phenolic and hydroxycinnamic acids, respectively [65]. The highest correlation was found between TEAC and RA content (r = 0.92). This correlation result suggests that the RA content in the IOSC extract has a high contribution and is more likely to be responsible for the antioxidant activity of IOSC extract for both elicitation or LAB fermentation treatments as was reported by Tepe [66]. It was found that RA was as expected topmost on the antioxidant activity of several Salvia plants. Additionally, Park *et al.* [1] highlighted that RA had a stronger antioxidant activity than that of vitamin E.

## 4. Conclusions

To conclude, from the selection of the high RA line, it was proven that the addition of yeast extract elicitor to the IOSC enhanced RA and the synthesis of phenolic compounds resulting in relatively higher antioxidant activity. A 70% methanol extract exhibited the highest extraction ability for antioxidant activity assays, total phenolics, flavonoids and flavonols. HPLC analysis of phenolic acids clearly confirmed a drastic increase in RA subjected to yeast extract elicitation. Therefore, this yeast extract elicited line of IOSC was used for the LAB fermentation study.

From the LAB fermentation study, the results clearly demonstrate that both types of fermentation, solid and liquid state fermentation, are deleterious to antioxidant properties and counterproductive for the retention of a high level of RA from harvested IOSC. The loss of antioxidant properties was more pronounced in the liquid state fermentation compared to the solid state one. Longer fermentation resulted in a greater reduction of the antioxidant properties. Results also reveal that the fermentation with *L. plantarum* resulted in the highest reduction rate of antioxidant properties. HPLC analysis confirmed that LAB fermentation of IOSC caused a decreased level of phenolic acids. Lastly, correlation analysis indicated that RA is the major contributor to the antioxidant activity of this plant.

## Supplementary Files

Supplementary File 1
