# Erratum: Hunaefi, D., *et al.* The Effect of *Lactobacillus plantarum* ATCC 8014 and *Lactobacillus acidophilus* NCFM Fermentation on Antioxidant Properties of Selected *in Vitro* Sprout Culture of *Orthosiphon aristatus* (Java Tea) as a Model Study. *Antioxidants* 2012, *1*, 4–32

**DOI:** 10.3390/antiox4040681

**Published:** 2015-10-29

**Authors:** Antioxidants Editorial Office

**Affiliations:** MDPI, Klybeckstrasse 64, Basel CH-4057, Switzerland; E-Mail: antioxidants@mdpi.com; Tel.: +41-61-683-77-35

## Introduction

Article [1] was published on 26 September 2012. In order to update the spelling of the second author’s name—“Divine N. Akumo” was written as “Divine Akumo”, it was updated a few days after publication and prior to issue release. However, an incorrect PDF version was inadvertently uploaded. This has recently been brought to our attention and we have replaced the PDF file and supplementary materials with the correct versions.

The Editorial Office takes full responsibility for this error. We apologize to the authors of the article and readers of the journal that this error was not fixed until now.
