# Supplementary material for: The Effect of Lactobacillus plantarum ATCC 8014 and Lactobacillus acidophilus NCFM Fermentation on Antioxidant Properties of Selected in Vitro Sprout Culture of Orthosiphon aristatus (Java Tea) as a Model Study
Source: Antioxidants (Basel). 2012 Sep 26;1(1):4–32. doi: 10.3390/antiox1010004 (PMC4665396; doi:10.3390/antiox1010004)
Supplement: Supplementary File 1 [file antioxidants-01-00004-s001.pdf]

OPEN ACCESS
antioxidants
ISSN 2076-3921
www.mdpi.com/journal/antioxidants

Supplementary Information

The Effect of *Lactobacillus plantarum* ATCC 8014 and *Lactobacillus acidophilus* NCFM Fermentation on Antioxidant Properties of Selected *in vitro* Sprout Culture of *Orthosiphon aristatus* (Java Tea) as a Model Study

Dase Hunaefi [1,2,]*, Divine Akumo [3], Heidi Riedel [1] and Iryna Smetanska [1,4]

[1] Department Method in Food Biotechnology, Institute of Food Technology and Food Chemistry, Berlin University of Technology, Königin-Luise Str. 22, 14195 Berlin, Germany; E-Mails: heidiriedel80@yahoo.de (H.R.); smetanska@mailbox.tu-berlin.de (I.S.)
[2] Department of Food Science and Technology, Bogor Agricultural University, Bogor, Indonesia
[3] Institute of Biotechnology, Laboratory of Bioprocess Engineering, Berlin University of Technology Ackerstr. 71-76, 13355 Berlin, Germany; E-Mail: akumo2@yahoo.com
[4] Department of Plant Food Processing, University of Applied Science Weihenstephan-Triesdorf, Steingruber Str. 2, 91746 Weidenbach, Germany

* Author to whom correspondence should be addressed; E-Mail: dase.hunaefi@mailbox.tu-berlin.de; Tel.: +49-30-314-712-63; Fax: + +49-30-832-766-3.

Tabel S1. Recent investigations on plant fermentation and its effect on antioxidant properties.

Plant	Condition of fermentation	Results			
		Phe	FD	FL	AA
White cabbage (*Brassica oleracea var. capitata* cv. Megaton) [1].	*L. plantarum* CECT 748, *Leuconostoc mesenteroides* CECT 219 or a mixed culture of both strains.	NA	NA	NA	Oxygen radical absorbance capacity (ORAC) values (up to 2-fold) and NO production inhibitory potency (up to 2.6-fold).
Oats (*Avena sativa* L.) [2]	SSF with *A. oryzae var. effuses*, *A oryzae*, and *A niger* on four subfractions of oats: n-hexane, ethyl acetate, n-butanol, and water with ethanol as solvent extractions.	Increased significantly ($p < 0.05$); e.g. oats water sub fraction from 1,580.1 ±62.6 mg GAE/100 g DW (un-fer.) to 3,632.7 ±73.1 mg GAE/100 g DW (fer. with *A. oryzae*).	Increased siginificantly ($p < 0.05$); e.g. Oats ethyl acetate sub fraction from 3,714.8 ±94.3 mg of rutin equivalents/100 g DW (un-fer.) to 7,893.1 ±397.3 mg of rutin equivalents/100 g DW (fer. with *A. oryzae*).	NA	Increased siginificantly ($p < 0.05$); e.g. Oats ethyl acetate sub fraction 747.5 ±14.6 micromoles of Trolox per gram of DW (un-fer.) to 1,687.9 ±40.7 (fer. with *A. oryzae*).

Tabel S1. *Cont.*

Plant	Condition of fermentation	Results			
		Phe	FD	FL	AA
Soybean [3]	The steamed soybeans were let stand for 1 h at 37 ℃ to cool down. After, the cooked soybeans was inoculated with 5% (w/w) strain *Bacillus subtilis* CS90 (1.43×10^7 cfu/mL) and fermented for 60 h at 37 ℃ in incubator and sampled at 0, 12, 24, 36, 48, and 60 h.	253 (0 h) increased to 9,414 mg/kg at the end of fermentation (60 h).	Increased from 53.43mg/kg (0 h) to 67.76 mg/kg (12 h)− 73.39 mg/kg (24 h) − 94.32 mg/kg (36 h) − 105.30 mg/kg (48 h) − 111.98 mg/kg (60 h).	Total flavonols increased (data divided into different type flavonols) although flavanol gallates contents decreased	The level of DPPH radical scavenging activity increased from 53.6 to 93.9% by 60 h.
Anoectochilus formosanus Hayata [4]	5×10^6 cfu/mL *L. acidophilus* BCRC 17002, *Bifidobacterium longum* BCRC 14602, *L. casei* subsp. *Casei* BCRC 12248 was inoculated into 100 mL vegetable juice.	Increased; e.g. leaf (un-fer.) 6.07 ±1.0 and fermented 14.05 ±1.0 mg/g.	NA	NA	It is clearly shown an increase in the detected antioxidant property may owe to the increase of total phenolic compounds.
Spirulina (*Arthrospira platensis*) [5]	*B.bifidum*, *L. casei*, *B. infantis*, *B. longum*, *Lactococcus lactis and L.acidophilus.*	The results of their study indicated that LAB-fermented Spirulina contained more polyphenols.	NA	NA	The greater quantity of total phenols in fermented samples indicates it possesses greater antioxidant activity.
Graptopetalum paraguayense E. Walther [6]	*L. acidophilus* BCRC 10695, *L. plantarum* BCRC 10357 and *L. paracasei* BCRC 14023.	Increased: e.g. water extract of immature *G. paraguayense* E. Walther fermentation by *L. plantarum* BCRC 10357 increased from 92.2 to 111 μg/mg.	Increased: e.g. water extract of immature *G. paraguayense* E. Walther fermentation by *L. plantarum* BCRC 10357 increased from 17.2 to 22.9 μg/mg.	NA	The level of antioxidants was significantly increased in immature *G. paraguayense* E. Walther fermented by *L. acidophilus* BCRC 10695, *L. plantarum* BCRC 10357 and *L. paracasei* BCRC 14023.

Tabel S1. *Cont.*

Plant	Condition of fermentation		Results			
			**Phe**	**FD**	**FL**	**AA**
Codonopsis lanceolata [7]	*Bifidobacterium longum* B6 and *L. rhamnosus* GG.		The fermentation process significantly increased the total phenol content of *C. lanceolata* when compared to the conventional extraction without fermentation. The total phenol content of *C. lanceolata* was the highest for high pressure assisted extraction from *L. rhamnosus* fermented (8.45 mg GAE/g), followed by *B. longum* fermented samples (8.25 mg GAE/g), non-fermented (7.38 mg GAE/g), and conventional extraction without fermentation (6.69 mg GAE/g).	Unlike the total phenols, fermentation decreased the total flavonoids. The lowest flavonoid contents were observed for high pressure assisted extraction of the fermented *C. lanceolata* with *B. longum* (0.44 mg RE/g) and *L. rhamnosus* (0.45 mg RE/g). The high pressure assisted extraction of un-fermented samples showed a maximum flavonoid content of 1.30 mg RE/g, followed by conventional extraction of un-fermented sample (0.78 mg RE/g).	NA	The lowest IC_{50} values were 1.25 mg/mL for high pressure assisted extraction of *B. longum* fermented sample and 1.18 mg/mL for *L. rhamnosus* fermented sample, indicating that the fermented *C. lanceolata* extract had the highest antioxidant properties.
Peanuts [8]	*Bifidobacterium longum* B17, *Lactobacillus casei* LC35 and *Lactobacillus acidophilus* LA51	Gallic acid, caffeic acid, chlorogenic acid and *p*-coumaric lactic acid fermentation resulted in a decrease in the content of these four phenolic acids.				Regardless of the starter organisms used, lactic acid fermentation could highly improve the DPPH radical-scavenging activity of PF (Peanut Flour). For example, at 2 mg/mL, the extract from FPF (Fermented Peanut Flour) showed 54.1 to 85.6% scavenging activity on DPPH radicals.

Phe = total phenolics; FD = total flavonoids; FL = total flavonols; andAA: antioxidant activity; SSF = solid state fermentations; and LSF = liquid state fermentations.

References

1. Martinez-Villaluenga, C.; Peñas, E.; Sidro, B.; Ullate, M.; Frias, J.; Vidal-Valverde, C. White cabbage fermentation improves ascorbigen content, antioxidant and nitric oxide production inhibitory activity in LPS-induced macrophages. *LWT-Food Sci. Technol.* **2012**, *46*, 77–83.

2. Cai, S.; Wang, O.; Wu, W.; Zhu, S.; Zhou, F.; Ji, B.; Gao, F.; Zhang, D.; Liu, J.; Cheng, Q. Comparative study of the effects of solid-state fermentation with three filamentous fungi on the Total Phenolics Content (TPC), flavonoids, and antioxidant activities of subfractions from Oats (*Avena sativa* L.). *J. Agr. Food Chem.* **2011**, *60*, 507–513.
3. Cho, K.M.; Lee, J.H.; Yun, H.D.; Ahn, B.Y.; Kim, H.; Seo, W.T. Changes of phytochemical constituents (isoflavones, flavanols, and phenolic acids) during cheonggukjang soybeans fermentation using potential probiotics *Bacillus subtilis* CS90. *J. Food Compos. Anal.* **2011**, *24*, 402–410.
4. Ng, C.-C.; Wang, C.-Y.; Wang, Y.-P.; Tzeng, W.-S.; Shyu, Y.-T. Lactic acid bacterial fermentation on the production of functional antioxidant herbal *Anoectochilus formosanus* Hayata. *J. Biosci. Bioeng.* **2011**, *111*, 289–293.
5. Liu, J.-G.; Hou, C.-W.; Lee, S.-Y.; Chuang, Y.; Lin, C.-C. Antioxidant effects and UVB protective activity of Spirulina (*Arthrospira platensis*) products fermented with lactic acid bacteria. *Process Biochem.* **2011**, *46*, 1405–1410.
6. Wu, S.-C.; Su, Y.-S.; Cheng, H.-Y. Antioxidant properties of *Lactobacillus*-fermented and non-fermented *Graptopetalum paraguayense* E. Walther at different stages of maturity. *Food Chem.* **2011**, *129*, 804–809.
7. He, X.; Zou, Y.; Yoon, W.-B.; Park, S.-J.; Park, D.-S.; Ahn, J. Effects of probiotic fermentation on the enhancement of biological and pharmacological activities of *Codonopsis lanceolata* extracted by high pressure treatment. *J. Biosci. Bioeng.* **2011**, *112*, 188–193.
8. Wang, N.-F.; Yan, Z.; Li, C.-Y.; Jiang, N.; Liu, H.-J. Antioxidant activity of peanut flour fermented with Lactic Acid Bacteria. *J. Food Biochem.* **2011**, *35*, 1514–1521.

© 2012 by the authors; licensee MDPI, Basel, Switzerland. This article is an open access article distributed under the terms and conditions of the Creative Commons Attribution license (http://creativecommons.org/licenses/by/3.0/).
